# Developments and clinical experiences in collagenase chemonucleolysis for lumbar disc herniation: a narrative review

**DOI:** 10.3389/fmed.2024.1522568

**Published:** 2025-01-08

**Authors:** Hao Zhang, Chi Zhang, Lin Li, Ming-liang Hu, Jian-ning Zhao, Zhang Zheng, Wen-feng Ding

**Affiliations:** ^1^Department of Radiology, Dianjiang People’s Hospital of Chongqing, Chongqing, China; ^2^Chongqing Yangjiaping Middle School, Chongqing, China; ^3^Department of Pharmacy, Dianjiang People’s Hospital of Chongqing, Chongqing, China; ^4^Department of Neurosurgery, Dianjiang People’s Hospital of Chongqing, Chongqing, China; ^5^Department of Radiology, Chongqing Traditional Chinese Medicine Hospital, Chongqing, China; ^6^Department of Orthopedic, Chongqing Kaizhou Guangming Orthopedic Hospital, Chongqing, China; ^7^Department of Orthopedic, Dianjiang People’s Hospital of Chongqing, Chongqing, China

**Keywords:** chemonucleolysis, oxygen-ozone, collagenase, CT-guided, lumbar disc herniation

## Abstract

Lumbar disc herniation (LDH) affects millions globally, with annual healthcare costs exceeding $100 billion in the United States alone, driving increasing interest in minimally invasive radiological interventions as treatment alternatives. This narrative review examines developments in collagenase chemonucleolysis for LDH, integrating a literature analysis with clinical experience. Key advancements include the transition from single-agent to combination therapies, exploration of diverse injection routes, and the progression from C-arm fluoroscopy to multi-slice CT guidance. The synergistic use of collagenase, oxygen-ozone, and anti-inflammatory analgesics has enhanced efficacy. Safety measures such as aspiration tests, contrast agent tests, and lidocaine tests implemented to mitigate procedural risks. However, challenges persist, including non-standardized dosages and potential complications arising from intradiscal injections. Future research should focus on establishing accreditation systems, refining patient selection criteria, optimizing drug dosages, and exploring advanced image-guided technologies. While chemonucleolysis offers advantages such as minimal invasiveness and cost-effectiveness, its complexity necessitates a multidisciplinary approach. Key findings demonstrate that combination therapy achieves superior outcomes compared to monotherapy, with long-term efficacy rates reaching 90% and 6-month success rates of 95%. Additionally, CT guidance has significantly improved procedural precision and safety compared to traditional fluoroscopy. This review provides insights for clinicians and researchers, highlighting the potential of chemonucleolysis in LDH management to ensure its safe and effective integration into mainstream treatment protocols.

## 1 Introduction

Lumbar disc herniation (LDH) is a prevalent spinal disorder that significantly affects patients’ quality of life, affecting approximately 1%–3% of the general population annually and being responsible for around 60% of low back and leg pain cases across diverse age groups, leading to over $100 billion in US healthcare expenditures per year (1, 2). Managing LDH is a daily challenge not only for medical doctors but also for chiropractors, physiotherapists, and traditional Chinese medicine practitioners. Each discipline employs a variety of approaches ranging from conservative therapies to more advanced interventional procedures. The management of LDH typically follows a stepped-care framework, which begins with conservative treatments and may escalate to minimally invasive procedures or surgical interventions if necessary (2, 3). Recently, the focus has increasingly shifted toward minimally invasive interventional treatments due to their ability to reduce recovery times and lower the risk of complications.

Within this context, chemonucleolysis using collagenase has gained attention as a compelling treatment option for LDH, effectively serving as a bridge between conservative management and more invasive surgical solutions. Collagenase chemonucleolysis was initially developed and applied in the United States during the 1960s–1980s, with studies reporting success rates of 70%–75% (4). However, despite FDA approval, its use declined in Western countries by the late 1990s due to multiple factors including safety concerns about allergic reactions, the emergence of microdiscectomy as a standard surgical option, commercial competition, and manufacturing issues, which led to a shift in preferences away from chemonucleolysis. Nevertheless, the development and refinement of chemonucleolysis continued in other regions, particularly in Asia. China approved collagenase as a Class I new drug in 1995 and gradually promoted its application (5). Condoliase (also known as chondroitinase ABC) has been approved in Japan since 2018, and multiple randomized controlled trials have demonstrated its efficacy and safety (6). This technique offers several benefits including minimal invasiveness, a favorable safety profile, established efficacy, and cost-effectiveness (7–9). It is a multidisciplinary approach that synthesizes insights from pain medicine, regional anatomy, radiology, and pharmacology, thus providing significant clinical value and opening new avenues for research. As chemonucleolysis continues to evolve, it embodies the collaborative efforts of various healthcare professionals, each bringing their unique expertise to enhance the efficacy and safety of this promising treatment.

The developments in collagenase chemonucleolysis have been characterized by continuous refinement and integration of complementary techniques. These advancements encompass diverse injection protocols, strategic use of contrast agents, improvements in imaging guidance technology, and enhancements in surgical risk management. These developments have expanded the treatment’s applicability while improving its overall efficacy and safety profile. This narrative summarizes the technique’s evolution and the existing issues in chemonucleolysis. By analyzing relevant literature and integrating our clinical experiences, we aim to provide clinicians with an integrated reference, promoting the further development and standardization of this technique.

To achieve this, we conducted an integrated literature search in PubMed, Web of Science, and CNKI (for Chinese literature) using the following strategy. We focused on articles published up to June 2024, prioritizing clinical studies and reviews that addressed collagenase for LDH treatment:

(Chemonucleolysis[MeSH] OR Chemonucleolysis[Title/Abstract]) AND (Oxygen-ozone[MeSH] OR Oxygen-ozone [Title/Abstract] OR Collagenases[MeSH] OR Collagenase [Title/Abstract]) AND (“Intervertebral Disc Displacement”[MeSH] OR “Lumbar disc herniation”[Title/Abstract] OR “Low Back Pain”[MeSH] OR Sciatica[MeSH]).

## 2 Pathophysiological basis, treatment mechanisms and clinical considerations

### 2.1 Pathogenesis of LDH

The pathogenesis of LDH has been extensively studied over the past century, with current understanding continuing to evolve. Three classical theories predominate regarding LDH pathogenesis:

1. Mechanical compression theory (10–12): Ruptures in both the inner and outer layers of the annulus fibrosus create fissures, allowing the protruding nucleus pulposus to directly compress or stretch the nerve root. This mechanical compression not only causes functional impairment of the nerve root but also leads to local tissue ischemia, edema, and demyelination. Under prolonged compression, these pathological changes can result in chronic nerve dysfunction, manifesting as lumbago and sciatica. The convex shape of the vertebrae makes mid and outer annular fibers particularly vulnerable to such mechanical stress, especially in the posterolateral region.
2. Chemical inflammation theory (11, 12): The nucleus pulposus contains various inflammatory mediators including phospholipase A2, histamine, lactate, bradykinin, substance P, calcitonin-gene related peptide, and vasoactive intestinal peptide. When these mediators come into contact with nerve roots due to herniation, they trigger the release of additional inflammatory factors, leading to chemical radiculitis and persistent pain. This inflammatory cascade can maintain pain signaling even after the mechanical compression has been relieved.
3. Autoimmune theory (10, 13): The nucleus pulposus, being an immune-privileged tissue normally sequestered from systemic circulation, can trigger autoimmune responses when exposed to the immune system through herniation. During the repair phase of annulus fibrosus lesions, newly formed capillaries infiltrate the nucleus pulposus tissue, allowing contact with immune cells. This exposure triggers both humoral and cell-mediated immune responses. Studies have detected specific immunoglobulin G (IgG) antibodies in degenerated discs, particularly reactive to matrix proteins like collagen II and aggrecan, with significantly higher autoantibody levels compared to non-degenerated IVDs.

**FIGURE 1:**
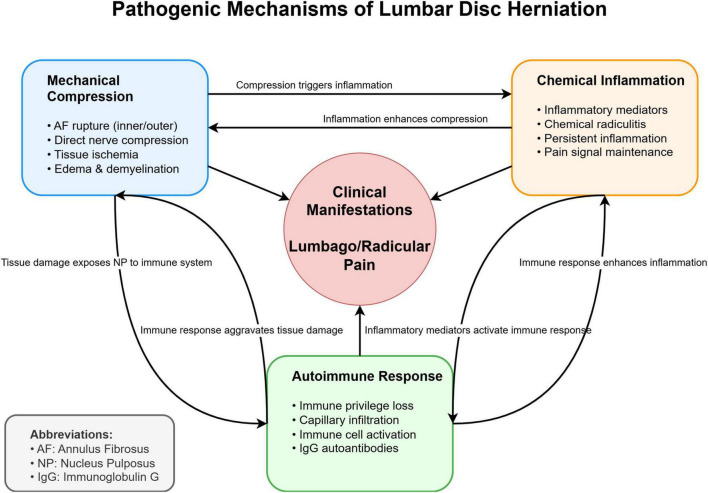
Schematic representation of the three major pathogenic mechanisms in lumbar disc herniation (LDH).

The interaction of these mechanisms collectively contributes to the clinical manifestations of LDH (Figure 1). While mechanical compression was historically considered the primary pathogenic factor, growing evidence suggests that chemical inflammation and autoimmune responses play crucial roles in both the initiation and maintenance of disc-related pain, particularly in cases where symptoms persist despite minimal mechanical compression (10, 11, 13).

The mechanical compression theory focuses on direct nerve root compression, the chemical inflammation theory emphasizes the role of inflammatory mediators, and the autoimmune theory highlights the immune response to exposed nucleus pulposus tissue. These mechanisms interact and collectively contribute to the clinical manifestations of LDH.

### 2.2 Mechanism of action of collagenase

Collagenase, a crucial component of chemonucleolysis, has been extensively studied for its mechanism of action since its isolation from *Clostridium histolyticum* by Mandl et al. (14). Research has demonstrated that collagenase possesses the following characteristics (8, 15):

(a) Specifically catalyzes the degradation of native, undenatured collagen;
(b) Effectively breaks down type I and II collagen fibers in the nucleus pulposus under normal physiological conditions;
(c) Degrades collagen into various amino acid molecules that can be absorbed by plasma;
(d) Forms an isotonic solution with human tissue and does not damage non-collagen protein substances, providing a wide safety margin.

When diluted in saline at room temperature, enzyme activity decreases by 75% after 6 h, necessitating on-site preparation for use. In degenerated discs, as the water content of the nucleus pulposus decreases, the collagen fiber content increases from the normal 20%–25% of dry weight to up to 60% (16). This increase enhances collagenase’s chemical ablation effect on the degenerated, protruding nucleus pulposus.

### 2.3 Treatment indications and patient selection

Treatment indications and contraindications are crucial considerations for successful chemonucleolysis implementation (3, 4). Chemonucleolysis alone is primarily indicated for contained disc herniations with radicular symptoms that have failed 1–4 weeks of conservative treatment. Combination therapy with ozone and anti-inflammatory analgesics is recommended for cases with significant inflammatory components or non-contained herniations. Contraindications include spinal cord injury or significant cauda equina symptoms, bony spinal stenosis or foraminal stenosis, spinal instability or spondylolisthesis, calcified or ossified disc material or posterior longitudinal ligament, allergies to collagenase, uncontrolled metabolic diseases, major organ dysfunction, coagulation disorders, uncontrolled infectious diseases, and psychological disorders incompatible with the procedure.

### 2.4 Efficacy assessment criteria

Treatment success was primarily defined as a reduction in pain scores (Visual Analog Scale or Numeric Rating Scale) by ≥50% or an absolute reduction of ≥2 points from baseline (17). Several studies utilized the Modified MacNab criteria, which classifies outcomes into four categories: “excellent” (complete resolution of symptoms, unrestricted daily activities), “good” (occasional non-radicular pain, return to modified work), “fair” (improved functional capacity but still handicapped), and “poor” (continued objective symptoms of root involvement, requiring further operative intervention). Treatment was considered successful when outcomes were rated as either “excellent” or “good” (6). Additionally, imaging changes (such as reduction in herniation size on MRI or CT) served as secondary outcome measures, although the correlation between imaging improvements and clinical outcomes varied.

## 3 Analysis of developmental trends

Since its introduction into clinical practice, chemonucleolysis has evolved in several key areas: (1) the exploration of multiple injection routes, (2) advancements in surgical techniques and drug combination strategies, (3) improvements in imaging guidance precision and contrast agent evaluation, and (4) the enhancement of safety protocols and risk management. These advancements have significantly enhanced treatment efficacy and substantially expanded treatment options for a wider range of cases.

### 3.1 Exploration of multiple injection routes

Currently, several injection routes have been developed (18, 19), including but not limited to:

(a) Combined intradiscal and extradiscal injection,
(b) Anterior epidural space injection via the intervertebral foramen,
(c) Injection through the sacral hiatus and posterior sacral foramina,
(d) Lateral recess injection,
(e) Targeted puncture injection at the medial edge of the small facet joint.

### 3.2 Evolution of technical approaches

**FIGURE 2:**
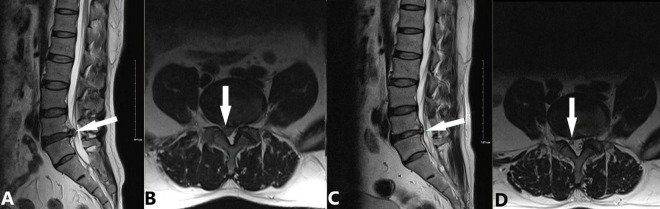
Application of targeted nucleus pulposus chemical ablation for LDH caused by extrusion at the right posterior margin of L4/5. A 45-year-old male patient presented with a 3-month history of lumbar distension and pain, which had progressively worsened in the past week. Considering the extruded nucleus pulposus and its moderate volume, we employed the CT-guided local targeted treatment method proposed by Wu et al. (8). The procedure involved the following steps: (1) A right posterolateral approach to puncture into the anterior epidural space; (2) Targeted injection at the extruded nucleus pulposus, including extradiscal injection of 600 U collagenase (WeiBang Biopharm, Liaoning, China) and 6.5 mL of an anti-inflammatory analgesic solution (0.5 mL betamethasone, 1 mL vitamin B_12_, and 5 mL 0.25% lidocaine); (3) No intradiscal injection of collagenase or oxygen-ozone mixture. **(A,B)** Preoperative images showing L4/5 disc protrusion (white arrow) compressing the dural sac and causing spinal stenosis. The right L5 nerve root is obscured by compression (**A**: sagittal view, **B**: axial view). **(C,D)** 90-day follow-up images showing complete resorption of the protrusion and decompression of the dural sac. The right L5 nerve root is clearly visible (white arrow; **C**: sagittal view, **D**: axial view).

**FIGURE 3:**
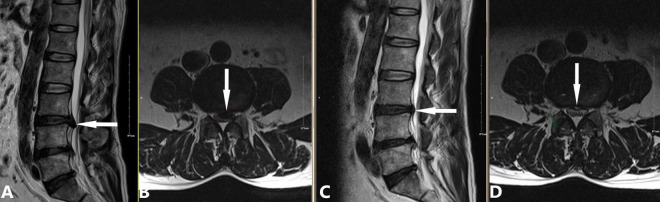
Case demonstration of the modified chemonucleolysis treatment for a large herniated nucleus pulposus. A 57-year-old male patient presented with a 5-year history of recurrent lower back distension and pain. Due to the large size of the herniated nucleus pulposus, we employed the modified treatment method proposed by Li et al. (20), consisting of the following steps: (1) Intradiscal injection of 10 mL oxygen-ozone mixture (40 μg/mL ozone); (2) Extradiscal injection of 1200 U collagenase and 6.5 mL of an anti-inflammatory analgesic solution; (3) No intradiscal collagenase administration. **(A,B)** Preoperative images showing L3/4 posterior disc protrusion (white arrow) compressing the dural sac and causing spinal stenosis (**A**: sagittal view, **B**: axial view). **(C,D)** 90-day follow-up images showing complete resorption of the protrusion and decompression of the dural sac (white arrow; **C**: sagittal view, **D**: axial view).

**FIGURE 4:**
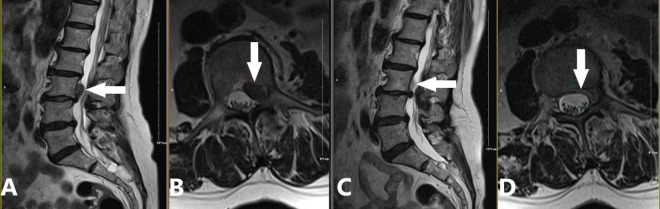
Application of the “three increases” treatment strategy for a sequestered disc fragment at the left posterior margin of the L3 vertebral body. A 69-year-old female patient presented with a one-month history of lumbar distension and pain. We applied a modified version of the “three increases” treatment strategy proposed by Zhang et al. (21), which included: (1) Increased puncture paths: Two coaxial needles at L2/3 (tip angled caudally) and L3/4 (tip angled cranially), creating a pincer effect on the L3 posterior margin extrusion; (2) Increased injection dose: 5 mL of oxygen-ozone mixture, 600 U of collagenase, and 5 mL of anti-inflammatory analgesic solution at each level, totaling 10 mL/1200 U/10 mL; (3) No increase in treatment frequency; (4) No intradiscal oxygen-ozone mixture or collagenase injection. **(A,B)** Preoperative images demonstrating a sequestered disc fragment (white arrow) originating from the L3/4 level and migrating superiorly to the left posterior margin of L3. The migrated fragment caused dural sac compression and spinal stenosis. Notably, there was no significant disc protrusion at the L3/4 level itself (**A**: sagittal view, **B**: axial view). **(C,D)** 90-day follow-up images demonstrating complete resorption of the sequestered disc fragment and decompression of the dural sac (white arrow; **C**: sagittal view, **D**: axial view).

These diverse injection routes offer more treatment options for managing various types and severities of LDH. The recommended strategy is via the intervertebral foramen through the left or right posterior approach, followed by either a combined intradiscal and extradiscal injection or an injection solely into the anterior epidural space. Researchers have made continuous improvements to chemonucleolysis techniques. These improvements have significantly enhanced the efficacy and applicability of chemonucleolysis in treating various types and severities of LDH. Key advancements include:

(a) 2009: Introduction of CT-guided targeted nucleus pulposus chemical ablation (8). This technique ensured safe and reliable collagenase injection while minimizing complications such as short-term increased intradiscal pressure and long-term intervertebral space narrowing (Figure 2).
(b) 2014: Development of a modified treatment method (20). This approach involved small-dose collagenase injection at an extradiscal target point of the lumbar intervertebral disc, combined with intradiscal oxygen-ozone injection for decompression. By leveraging the complementary advantages of oxygen-ozone and collagenase, this method achieved a success rate of 90.0% in treating massive LDH, overcoming traditional contraindications of chemonucleolysis (Figure 3).
(c) 2016: Proposal of the “three increases” treatment strategy (21). This strategy involved increasing collagenase injection dose, expanding injection routes, and raising treatment frequency, further broadening the application of collagenase treatment for LDH and offering new options for severe cases (Figure 4).

### 3.3 Evolution and synergistic effects of combination therapy

Chemonucleolysis for LDH treatment has transitioned from single-agent approaches to more sophisticated combination therapies. This evolution occurred in three stages:

(a) Monotherapy Era: Initially, chemonucleolysis relied on single-agent injections, primarily collagenase or oxygen-ozone. A meta-analysis of 897 patients demonstrated significant efficacy for both oxygen-ozone and collagenase monotherapies in LDH management (22).
(b) Introduction of adjunct therapy: Subsequent studies revealed that administering a small amount of mixed solution—primarily corticosteroids and local anesthetics—following ozone or collagenase injection could enhance therapeutic efficacy (23–25). This mixture is termed the “Anti-inflammatory and Analgesic Solution” based on its action and effectiveness.
(c) Integrated combination therapy: As clinical experience and research progressed, the combined application of collagenase, oxygen-ozone, and the anti-inflammatory analgesic solution emerged as the predominant treatment modality. A meta-analysis (17) demonstrated superior efficacy of this combined protocol compared to collagenase chemonucleolysis alone. The long-term efficacy of this integrated approach reached 90%, with a stable success rate of 95% at 6-month follow-up.

This integrated approach offers synergistic advantages: (a) short-term: rapid symptom relief from oxygen-ozone and the anti-inflammatory analgesic solution; (b) long-term: collagenase directly acts on the herniated material, achieving decompression. This dual-action strategy not only improves clinical outcomes but also potentially reduces the need for repeated interventions, offering a more integrated, sustained, and patient-friendly treatment option for LDH management. It arises from the unique mechanisms of action of its individual components. Oxygen-ozone plays a key role in reducing inflammation and decompression, while the anti-inflammatory and analgesic solution enhances pain relief and tissue recovery. The following sections provide a detailed examination of these mechanisms.

#### 3.3.1 Mechanism of action of oxygen-ozone

Oxygen-ozone therapy for LDH has gained significant traction in recent decades, with numerous studies supporting its efficacy. Muto and Avella (26) reported a 78% efficacy rate for oxygen-ozone injections into the intervertebral disc and paravertebral epidural space. Alexandre et al.’s (27) multicenter study in Italy showed an 80.9% success rate and an overall efficacy rate of 93% for oxygen-ozone therapy. Research has revealed the multifaceted mechanisms of action of oxygen-ozone (20, 26, 27):

(a) Strong oxidation: Oxygen-ozone exhibits an oxidation rate 300–600 times higher than that of oxygen, effectively decomposing proteins and polysaccharide macromolecular polymers within the nucleus pulposus.
(b) Decompression effect: It induces water loss, contraction, degeneration, and necrosis of nucleus pulposus tissue, reducing intradiscal pressure, promoting annulus fibrosus retraction, and alleviating nerve root compression.
(c) Anti-inflammatory and vascular effects: It stimulates vascular endothelial cells to release nitric oxide and platelet-derived growth factor, promotes vasodilation, improves venous return, and inhibits local immune responses, reducing nerve root edema.
(d) Mechanical effect: It creates physical separation at the puncture site, breaking inflammatory adhesions and facilitating better distribution of subsequent medication, thereby enhancing therapeutic efficacy.

These mechanisms collectively contribute to the overall efficacy of oxygen-ozone therapy in LDH treatment. The half-life of oxygen-ozone under normal conditions is 22.5 min, requiring on-site preparation for immediate use.

#### 3.3.2 Mechanism of anti-inflammatory and analgesic solution

Following the administration of oxygen-ozone and collagenase, the introduction of an anti-inflammatory and analgesic solution, consisting of precise proportions of adenosine cobamide or vitamin B_12_, betamethasone, and lidocaine, has been shown to elicit multiple therapeutic effects (20, 23–25):

(a) Dilates capillaries and improves local microcirculation
(b) Expands the perineural space and separates nerve root adhesions
(c) Dilutes inflammatory mediators and alleviates tissue edema and exudation
(d) Rapidly reduces inflammation and pain, disrupting the vicious cycle of pain conduction

### 3.4 Advancements in imaging-guided technology

The evolution from early C-arm fluoroscopy to current multi-slice spiral CT (MSCT) has significantly enhanced the precision and safety of imaging-guided procedures. While C-arm fluoroscopy rapidly offers real-time imaging, it has several limitations (21, 28):

(a) It only produces overlapping images of the lumbar spine and is unable to visualize soft tissue structures such as intervertebral discs, dural sacs, and nerve roots.
(b) Operators are required to wear heavy protective clothing, which increases the risk of vascular and neural injury during needle insertion.
(c) The process of rotating the X-ray tube to obtain anterior-posterior and lateral radiographs for confirming needle path and target is time-consuming.

**FIGURE 5:**
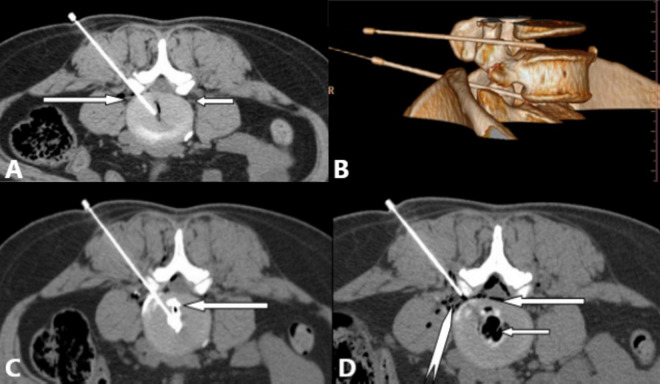
Multi-slice spiral CT provides high-resolution, non-overlapping axial images for precise puncture; offers multiplanar reconstruction (MPR) and volume rendering (VR) capabilities for rapid, accurate guidance; and allows evaluation of lumbar annular fissures and oxygen-ozone distribution. **(A)** Right posterolateral approach for L4/5 intradiscal treatment. White long arrow: left L5 nerve root; white short arrow: right L5 nerve root. **(B)** VR 3D reconstruction showing puncture paths (safe triangle area) for L4/5 (white short arrow), and L5/S1 (white long arrow) discs. **(C)** L4/5 discography revealing single lumbar annular fissures (white long arrow) and contrast agent distribution, guiding treatment planning. **(D)** Needle position for epidural anterior space treatment post-intradiscal therapy at L4/5. White long arrow: epidural oxygen-ozone distribution; white short arrow: intradiscal oxygen-ozone distribution; white dovetail arrow: L5 nerve root.

With its technological advancements, MSCT has been widely adopted, offering several advantages (20, 21) (Figure 5):

(a) High-definition, non-overlapping cross-sectional images for clear visualization of target discs and adjacent structures.
(b) Integration of multi-planar reconstruction (MPR) for accurate measurement of puncture angles and depths, enabling safer and more efficient procedures.
(c) Reduced radiation exposure for both patients and medical staff.

A study of 78 LDH cases treated with CT-guided oxygen-ozone injection (29) detailed the impact of five ozone distribution patterns on treatment outcomes, an analysis impossible with C-arm fluoroscopy. Another comparative study (30) demonstrated CT’s superiority in reducing complications, improving procedural efficiency, puncture accuracy, and overall efficacy.

### 3.5 Application of contrast agents

Contrast agents play a crucial role in enhancing the precision and efficacy of LDH treatments:

Epidural and nucleus pulposus visualization: (a) Injecting a small amount of contrast agent through a coaxial puncture needle helps visualize the distribution in the epidural space and nucleus pulposus protrusion. (b) This guides operators in adjusting the needle path and position for optimal drug distribution (4).

Discography for annulus fibrosus assessment: (c) Injecting contrast agent into the intervertebral disc reveals the type of annulus fibrosus tear (contained, protruded, or ruptured). (d) This information guides the selection, concentration, and dosage of injected drugs to enhance efficacy.

The DDD (Dallas Discogram Description) system (31) aids in designing individualized treatment plans. For example: (a) Full-thickness annulus fibrosus tear (DDD grade 5): this pattern suggests that the injected solution enters the posteriorly protruding nucleus pulposus tissue through a single annular fissure. In this scenario, intradiscal injection of a small amount of collagenase may be considered to enhance treatment efficacy. (b) Diffuse annulus fibrosus tears (DDD grade 7): multiple annular fissures make it unlikely that intradiscally injected collagenase will specifically target the protruding nucleus pulposus. Consequently, collagenase injection should be avoided to prevent excessive disc dissolution and subsequent long-term intervertebral space narrowing.

### 3.6 Procedural risks and safety measures

In addition to common complications such as allergic reactions and infections, chemonucleolysis carries a significant risk of inadvertent leakage or injection of the chemonucleolytic agent into the subarachnoid space. This may occur due to procedural errors and, if not promptly detected or properly managed, can lead to chemical meningitis. In severe cases, this complication may cause neurological deficits or even death (31, 32). Case reports suggest that timely cerebrospinal fluid replacement and lavage with physiological saline to dilute the misplaced agent may mitigate the damage (32).

To mitigate this risk, three key preventive measures are implemented:

(a) Aspiration test: Performed prior to drug injection to ensure the needle tip is not positioned within a blood vessel or the cerebrospinal fluid-containing subarachnoid space (33).
(b) Contrast agent test: Conducted after needle placement to detect any potential dural sac damage by injecting contrast medium and observing its distribution (4, 20).
(c) Lidocaine test: Administered prior to collagenase injection to exclude intraspinal anesthesia. A 1% lidocaine solution is initially administered, and the patient is monitored for 5 min, with lower limb reflexes, sensation, and muscle strength assessed (20).

These preventive measures have significantly reduced complication rates. However, strict adherence to protocols and constant vigilance remain crucial. Notably, the aspiration test, a standard procedure for all injections, prevents inadvertent administration of agents into blood vessels or the subarachnoid space, such as the potentially life-threatening gas embolism from accidental intravascular oxygen-ozone injection.

## 4 Existing issues

### 4.1 Drug dosage and standardization challenges

The total extradiscal collagenase dose commonly used in China is 1,200 U, but intradiscal injection doses vary, ranging from 400 to 1,200 U (5, 33). Studies comparing the efficacy of 400 and 1,200 U collagenase for LDH treatment (34), and another comparing 1,200 and 2,400 U via epidural lateral recess injection (35), found no significant difference in outcomes despite substantial dose variations. This aligns with the Michaelis–Menten equation (36), where reaction rates plateau when enzyme concentration exceeds substrate levels, resulting in a non-linear dose-effect relationship. Additionally, reported collagenase dilution protocols vary, with 600 U dissolved in 1, 2, 3, or 6 mL of saline (6, 30). Similarly, medical oxygen-ozone concentrations range from 20 to 60 μg/mL (32, 37), with no standardized protocol.

### 4.2 Impact of drug combinations on collagenase activity

Conflicting views exist regarding the impact of drug combinations on collagenase activity:

(a) One study (38) suggests that dexamethasone (a corticosteroid), lidocaine (an anesthetic), and iopamidol (a contrast agent), either alone or in combination, inhibit collagenase activity to varying extents. This study recommends avoiding mixed administration to maintain efficacy.
(b) Another study (39) partially supports this view, confirming lidocaine’s inhibitory effect on collagenase activity. However, it found that dexamethasone mixed with collagenase did not significantly affect its activity. Interestingly, the combination of lidocaine and dexamethasone may negate lidocaine’s inhibitory effect.

These inconsistent findings have led to a lack of consensus in China regarding the use of anti-inflammatory and analgesic solutions in chemonucleolysis. As a result, some practitioners opt to avoid these combinations altogether.

### 4.3 Intradiscal injection and associated complications

While effective, intradiscal injection of collagenase may lead to several complications (5, 40):

(a) Excessive dissolution of the intervertebral disc: This can result in significant loss of disc material, potentially leading to spinal instability.
(b) Endplate inflammation secondary to endplate injury: This inflammatory response can cause persistent pain and potentially affect adjacent vertebral bodies.
(c) Loss of disc height: This occurs due to excessive degradation of the disc matrix, potentially altering spinal biomechanics.
(d) Secondary foraminal stenosis: The loss of disc height can lead to foraminal narrowing, potentially causing nerve root compression and associated radicular pain.

**FIGURE 6:**
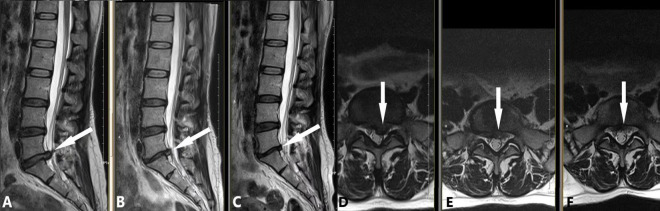
An early chemonucleolysis case demonstrating long-term follow-up and potential complications. A 48-year-old female patient presented with a 2-week history of bilateral sciatica and 1-week history of perianal heaviness. This early case, performed during our initial experience, followed this strategy: (1) Intradiscal injection of 10 mL oxygen-ozone mixture and 200 U collagenase; (2) Extradiscal injection of 1000 U collagenase and 6.5 mL anti-inflammatory analgesic solution. MRI findings are as follows: **(A,D)** Preoperative images showing L5/S1 posterior disc protrusion (white arrow; **A**: sagittal view, **D**: axial view) compressing the dural sac. **(B,E)** 90-day follow-up images demonstrating significant protrusion resorption (white arrow; **B**: sagittal view, **E**: axial view) with L5/S1 intervertebral space narrowing. **(C,F)** 180-day follow-up images showing further protrusion reduction (white arrow; **C**: sagittal view, **F**: axial view), with L5/S1 space narrowing unchanged from the previous exam.

These complications may cause patients to experience pain during the postoperative period or long-term follow-up. Given these risks, the intradiscal injection technique is currently less frequently employed in clinical practice (Figure 6). However, when performed with proper technique and patient selection, chemonucleolysis can still be an effective treatment option with a relatively low complication rate compared to more invasive surgical procedures (6). The 2024 systematic review by Schol et al. (6) demonstrated a 79% overall treatment success rate for chemonucleolysis, with the procedure significantly outperforming placebo controls (OR 3.35, 95% CI 2.41–4.65) and showing comparable efficacy to surgical interventions (OR 0.65, 95% CI 0.20–2.10). The study also reported a low severe adverse event rate of only 1.4% across 12,368 patients (6).

## 5 Future prospects

Collagenase chemonucleolysis for LDH treatment offers significant advantages, including minimal invasiveness, rapid onset of action, shorter hospital stays, lower medical costs, and reduced complication rates, all of which make it readily acceptable to patients. When operators strictly adhere to indications, correctly execute surgical procedures, and uphold the principle of “drug to the lesion, enzyme to the substrate,” chemonucleolysis can become an important option for LDH patients, warranting further clinical application and in-depth research.

Nevertheless, chemonucleolysis requires multidisciplinary knowledge, including pain medicine, local anatomy, pharmacology, radiology, and non-vascular interventional techniques, necessitating a cautious approach to its future development. Given the complexity of the disease, the technical intricacies of the procedure, and potential complications, future research should focus on:

(a) Establishing relevant accreditation systems to standardize operator qualifications.
(b) Clarifying patient selection criteria for different types and sizes of disc herniations.
(c) Further quantifying and refining drug dosages to optimize treatment efficacy and minimize risks of adverse reactions.
(d) Exploring new image-guided technologies, such as robot-assisted localization, to enhance surgical precision and safety.

## 6 Limitations

This narrative review has several limitations that warrant acknowledgment.

(a) First, the predominant application of collagenase chemonucleolysis in Asian countries has resulted in a significant portion of the cited literature being in Chinese, with relatively few English-language publications. This linguistic imbalance may introduce potential bias in the comprehensiveness of our review and limit its generalizability to global practice patterns. Nevertheless, it also provides a window for mutual learning across different regions, offering insights into practices that may not be widely disseminated in English-language journals.
(b) Second, the authors’ clinical experience and expertise are primarily focused on the current technique of collagenase chemonucleolysis. Consequently, we lack comprehensive understanding and hands-on experience with other minimally invasive treatments for LDH. This limitation precludes us from conducting a thorough comparative

analysis of various minimally invasive techniques for LDH management. While we have endeavored to present an objective overview, our perspective may be influenced by our specific clinical focus.

## 7 Conclusion

In conclusion (Table 1), through continuous clinical practice and exploration, collagenase chemonucleolysis has achieved significant advancements in LDH treatment. Looking ahead, a balanced approach that combines innovation with caution is essential to refine this technique and ensure its safe and effective integration into mainstream LDH treatment protocols.

**TABLE 1:** Overview of key developments.

Aspect	Key developments
Injection routes	Development of various methods, including combined intradiscal and extradiscal injections, and anterior epidural space injection via the intervertebral foramen
Surgical technique improvements	Introduction of CT-guided targeted nucleus pulposus chemical ablation, modified treatment methods, and the “three increases” treatment strategy
Treatment modality evolution	Evolution from single collagenase injection to combined application with oxygen-ozone and anti-inflammatory analgesic solution
Image-guided technology	Progression from C-arm fluoroscopy guidance to multi-slice spiral CT (MSCT) guidance, enhancing precision and safety
Contrast agent application	Utilization for assessing annulus fibrosus tear types, guiding personalized treatment plans
Existing issues	Non-standardized drug dosages, drug compatibility issues, and complications related to intradiscal injection
Future prospects	Establishing accreditation systems, refining patient selection criteria, optimizing drug dosages, and exploring advanced image-guided technologies
